# Job Demands, Resources and Burnout Among Polish Nurses During the Late Wave of COVID-19 Pandemic: The Mediating Role of Emotional Labor

**DOI:** 10.3389/fpsyt.2022.931391

**Published:** 2022-07-08

**Authors:** Grzegorz Wójcik, Antoni Wontorczyk, Ilona Barańska

**Affiliations:** ^1^Department of Medical Sociology, Faculty of Medicine, Jagiellonian University Medical College, Kraków, Poland; ^2^Institute of Applied Psychology, Faculty of Management and Social Communication, Jagiellonian University, Kraków, Poland

**Keywords:** JD-R model, nurses, emotional labor, burnout, Poland, COVID-19

## Abstract

**Objective:**

Burnout has been recognized as a serious health problem. Nurses as a professional group are at a high risk of burnout occurrence, especially when facing burden associated with the COVID-19 pandemic. Despite evidence that higher job demands lead to burnout, there is less known about the indirect effect of job demands and resources on burnout via surface acting. Using the JD-R framework, this study examined how job demands and resources affected burnout among Polish nurses and whether these relationships are mediated by surface acting and moderated by coping with the workload.

**Materials and Methods:**

A sample of 270 nurses from the biggest hospital in Southern Poland filled out an online questionnaire at the time between the fourth and the fifth wave of the COVID-19 pandemic in Poland. The Polish adaptations of Oldenburg Burnout Inventory (OLBI), Organizational Constraints Scale (OCS), Interpersonal Conflict at Work Scale (ICAWS), Areas of Worklife Survey (AWS), and Emotional Labor Scale (ELS) were used. Mediation and moderation analyses were carried out in the SPSS macro-PROCESS.

**Results:**

Surface acting partially mediated the positive association between organizational constraints and interpersonal conflict at work and burnout, as well as the negative association between the perceived organizational support and burnout. Coping with workload moderated the direct effect of organizational constraints on burnout via surface acting.

**Conclusion:**

The findings enrich the knowledge of the mediating and moderating mechanisms to explain the association between job demands, resources and burnout among nurses. There have been proposed interventions concerning increasing organizational support, effective emotional regulation of management education and psychological training regarding adequate coping strategies which could help reduce or prevent the occurrence of burnout in this professional group.

## Introduction

The World Health Organization (WHO) recognizes occupational burnout as a result of chronic stress experienced in the workplace consisting of three dimensions: the feeling of loss, negative feelings concerning work and the mental distance toward it, as well as reduced professional effectiveness (1). Nurses as a professional group are highly subject to burnout due to, first of all, direct contact with patients who require emotional involvement, while also dealing with various possible situations, including the patients’ suffering, fear or aggression (2, 3) and secondly, the relatively low position in the working organization such as a hospital and tasks they are generally assigned to Lasalvia et al. (4). Thus, nurses are known to be at higher risk for the development of burnout compared to other medical occupations (3). A study that examined the prevalence of burnout among 1,329 medical professionals from five different professional fields found that nurses experience its highest level (5). Similarly, the results of a systematic review of Khamisa et al. (6) showed that high levels of work-related stress, burnout, and poor health are common among nurses. Woo et al. (7), in turn, based on 113 studies included for systematic review and 61 studies considered in the meta-analysis showed that 11.23% of nurses worldwide experienced burnout symptoms.

The situation of Polish nurses in terms of burnout seems to be even worse as the study of Uchmanowicz et al. (8) reported increasing levels of burnout and worsening job satisfaction among members of this professional group. Additionally, Poland has one of the lowest indicators of practicing nurses per 1,000 residents among the OECD countries. In 2015, it was by comparison only 5.2; whereas this indicator for Germany was 13.3, Denmark–16.7 and Switzerland–18 (9). The high average age of nurses in Poland was estimated to be around 52 years of age, which, when combined with the high percentage of nurses dissatisfied with their working conditions leads them to the feeling of work overload and high levels of occupational stress and burnout (10, 11).

The already high prevalence of burnout among nurses in ordinary times became an even more serious concern during the current COVID-19 pandemic due to the emergence of new stressors at their workplace, such as the high death rate of COVID-19 patients, the increased number of overtime shifts, the fear of not getting the appropriate medical equipment (including personal protective equipment) or the possibility of COVID-19 transmission and the infection of family members (12, 13). According to a systematic review by Galanis et al. (14), nurses have experienced high levels of burnout during the COVID-19 pandemic, as the overall prevalence of emotional exhaustion in this professional group led to 34.1%, while depersonalization amounted to 12.6% and the lack of personal accomplishment totaled 15.2%.

According to the Job Demands-Resources theory (JD-R), occupational burnout is a long-term effect of stress caused by prolonged excessive job demands and insufficient resources to deal with these job demands effectively (15–18). Burnout in this perspective consists of two components, named exhaustion and disengagement from work (19). The exhaustion dimension refers to feelings of physical fatigue and overload with relation to work (20), while the dimension of disengagement refers to the distance from work and negative attitudes toward their own work (19). In addition, it is assumed that job resources buffer the potentially negative effects of excessive job demands on employee health and well-being (21, 22).

Different studies conducted among nurses which applied JD-R theory confirmed that such stressors, as excessive workload (23, 24) third-party aggression (23, 25), emotional and organizational demands (26, 27) are related to burnout among this professional group. However, the amount of research which used JD-R model to examine direct relationships as well as indirect via emotional labor between such fundamental stressors for nursing staff as organizational constraints and interpersonal conflict at work and burnout is scarce (28, 29). Thus, our study is an attempt to fill this gap in the scientific literature.

Organizational constraints are related to situations or things that prevent employees from translating ability and effort into a high level of job performance. These include faulty equipment, incomplete or poor information flow, as well as interruptions by others (30). Garcia-Arroyo et al. (31) demonstrated that organizational constraints considered from the JD-R perspective should be regarded as demands that can limit autonomy, job control, and decision-making capacity, generating stress, and burnout. Similarly, using JD-R framework, Baka (32) showed that organizational constraints were associated with job burnout and depression among Polish teachers. In turn, interpersonal conflicts at work are defined as a negative interpersonal encounter disturbing team cohesion and characterized by a contentious exchange, hostility or aggression (30). This demand has been acknowledged as a persistent problem and one that is on the rise among nursing personnel (33). In the study of Lanz and Bruk-Lee (34) conducted among US nurses, interpersonal conflict at workplace predicted both burnout and turnover intention. Similarly, interpersonal conflicts were found to be positively related to emotional exhaustion, depersonalization and negatively in terms of personal accomplishment among Emergency Room-nurses in Spain (35).

In the current study interpersonal conflicts are considered in the context of theory of social relations which seems to be relevant within the context of hospital as a work organization (36). Interpersonal conflict with coworkers is likely to affect adversely nurse’s psychological health because relationships among coworkers are based on a communal sharing model of interpersonal relations. In turn, interpersonal conflict with nurse’s supervisor leads to negative feelings and cognitions regarding one’s job because an employee’s relationship with supervisor is based on an authority ranking model in the Fiske’s theory (36). In the current study, we do not distinguish conflicts regarding coworkers or conflict between worker and supervisor and treat them together. Both organizational constraints and interpersonal conflicts at work are viewed as hindrance stressors because they serve as barriers for nurses to goal accomplishment (37). Based on mentioned studies, we formulate hypothesis 1:

***H1:*** *Job demands will have direct effects on burnout with organizational constraints and interpersonal conflict at work having a positive association with burnout.*

With regard to job resources, social support has been acknowledged as an important resource which influences of an individual’s perception of threatening situation at work (38). Organizational support has been recently recognized as a crucial job resource in nursing staff (39). The high level of organizational support has been acknowledged as a factor which reduced burnout among nurses in China (40). Moreover, Escriba-Aguir and Pe’rez-Hoyos (41) reported that low supervisory social support was related to higher emotional exhaustion in nurses, whereas in their longitudinal study, Van der Ploeg and Kleber (42) found the lack of supervisory social support at the baseline to be related to higher emotional exhaustion and depersonalization, while also to lower personal accomplishment in the follow up. Accordingly, we present hypothesis 2:

***H2:*** *Organizational support as a job resource will be directly negatively associated with burnout.*

Baka and Prusik (24) indicated that currently researchers who apply JD-R model are looking for potential mediational variables that would deepen the understanding of mechanisms regulating the relationship between job demands and burnout. One of them is emotional labor which recently has been attracting attention as a factor that relates to burnout and turnover intention among nurses (43). Emotional labor refers to the effort involved in managing feelings when the work role specifies that particular emotions should be displayed and others should be hidden (44). Healthcare staff, especially nurses, are required to deliver compassionate care to the patients in frequently challenging working conditions. However, demands of emotional labor occur when a nurse has to change her actual emotions in order to present emotions that conform to the rules and expectation of the job (44). In the case of demanding and stressful situations such as organizational difficulties or interpersonal conflict at work, nurses adopt surface acting in order to display the emotions required for their role, albeit their true feelings remain unchanged (emotional dissonance) or their negative feelings are simply suppressed (emotional suppression) (45, 46). Since engaging in surface acting requires higher levels of self-control, this kind of emotional labor has been found to be significantly associated with burnout and depressive symptoms among nurses (47). For example, the study conducted by Gilardi et al. (25) which applied JD-R framework, revealed that surface acting mediated the relationship between patients and/or their relatives’ aggression and burnout among nurses in Italy. In line with that research, we decided to focus especially on surface acting as a detrimental process leading to burnout among nurses. We would like to distinguish factors which are positively and negatively related to surface acting in order to broaden knowledge concerning this kind of emotional labor and, in effect, gain an opportunity to counteract it. We assume that organizational constraints and interpersonal conflict at work as hindrance stressors provoke emotional dissonance/emotional suppression considered as surface acting because the negative emotions that are triggered, may be appraised as inconsistent with the interiorized role of nurse as an empathetic healthcare worker (48). Thus, in the next hypothesis we expect that:

***H3:*** *Job demands will indirectly affect burnout via surface acting, indicating the mediating effect of surface acting on the relation between organizational constraints, interpersonal conflict at work and burnout.*

With respect to the relationship between perceived organizational support and emotional labor, previous studies demonstrated that the high level of organizational support leads to high levels of effort in deep acting, whereas in the case of feeling a low level of organizational support, nurses simply choose a less effortful way, i.e., surface acting, to perform emotional labor (49, 50). Therefore, we expect that higher perceived organizational support will be associated with lower surface acting and, in turn, with lower burnout level among nurses. Accordingly, we present hypothesis 4:

***H4:*** *Surface acting will mediate the relationship between organizational support and burnout.*

Apart from job resources, the JD-R model recognizes personal resources, for instance coping strategies, which are expected to buffer the undesirable impact of job demands on strain (21). Coping behavior is defined as constantly changing cognitive and behavioral efforts to manage specific external or internal demands that are appraised as exceeding the resources of the person (51). In the current study, coping with workload has been considered as a kind of proactive coping method defined as self-determined goal setting behavior that motivates people to overcome difficulties in order to achieve desirable personal outcomes and growth. In essence, proactive coping consists of the accumulation of various resources and the attainment of skills such as organization, planning, goal-setting, and mental simulation (52). Negative association between proactive coping and burnout has been reported in previous studies (53, 54). Moreover, the buffering effect of proactive coping on relationship between work stress and burnout has been confirmed in the study of Greenglass (55). Thus, in our last hypothesis we expect that:

***H5:*** *Coping with workload will moderate the direct path in the mediation process of organizational constraints on burnout through surface acting. These relations will be weaker for nurses with higher capabilities of coping with workload.*

### The Late Wave of COVID-19 Pandemic in Poland and Nursing

There are no studies exploring the burnout level among Polish nurses specifically during COVID-19 pandemic so far. However, according to the research by Szwamel et al. (56) which encompassed all healthcare workers in Poland, 71.63% (356) of the respondents presented high and moderate levels of emotional exhaustion, 71.43% (355) reported low and moderate job satisfaction levels, whereas 40.85% (203) displayed high and moderate levels of depersonalization. The current research has been conducted during the late wave of COVID-19 pandemic, which means that the organization of work of nurses and the level of job demands were different from traditional ones (57).

The late wave of COVID-19 pandemic in Poland has taken place in the end of 2021 and at the beginning of 2022. Despite of full access to vaccination, the situation paradoxically seemed to be quite dramatic, especially because of very high rate of death due to COVID-19. For example, on 29 December has been reported the highest rate of death during the whole pandemic period – 795 persons passed away this day (58). Moreover, since January until February 2022 the number of confirmed cases with COVID-19 has increased four times, i.e., from 11,775 on 5th January to 45,800 cases reported on 5th February. However, the number of hospitalizations due to COVID-19 during this period decreased (59). There is insufficient amount of research conducted during the late wave of pandemic concerning mental health of nurses therefore our study could be regarded as a contribution to the current knowledge.

## Materials and Methods

### Sample and Data Collection

This cross-sectional study was conducted in the University Hospital of Cracow, the largest hospital in Southern Poland. The survey was conducted from 3 January to 6 February 2022. An online survey was created using the Qualtrics platform and link for the study, which was sent to all hospital nurses. The inclusion criteria applied related to a nurse and female gender of the respondent. Participation in the research was completely voluntary and anonymous.

At the beginning of the survey, the participants were informed about the general purpose of the study, while also the fact that the study is non-invasive and the results would be analyzed anonymously. It was also explained that they could withdraw from the study by closing the web browser without their responses being recorded. The participants provided their written consent prior to participating in this study. The contact details of the person responsible for the project was provided in case they wished to obtain additional information or had any questions concerning the study.

The final research group consisted of 311 nurses. Due to incomplete responses, or the failure to meet the inclusion criteria, 41 people were removed from the analysis. The complete data obtained from 270 participants was then included in the statistical analysis. The average completion time of the survey was 20 mins. To calculate the sample size for the study we used the formula: *n* ≥ Z^2*^p(1 − *p*)/d^2^, where *n* is size sample, *Z* is the z score for α = 0.05 (=1.96), *p* is prevalence of disease in population and *d* is the margin of error. We included a correction for a finite population in the calculations. For this, we used formula: n_adj_ = Nn/(*N* + *n* − 1) (60). Based on general sample size (total number of nurses) of 1,789, prevalence of burnout during the COVID-19 pandemic among nurses of 0.2 (61), 5% margin of error, and 95% confidence level, the optimal sample size for this study was calculated at 216 nurses.

### Measures

#### Burnout

The Polish adaptation (62) of the Oldenburg Burnout Inventory – OLBI (19) was applied. This 16-item scale was divided into two subscales - exhaustion (e.g., “There are days when I feel tired before I arrive at work”) and disengagement from work (e.g., “It happens more and more often that I talk about my work in a negative way”). Each subscale contains eight items, which are worded in a positive way (four items) and a negative way (four items). Exhaustion encompasses both cognitive and physical fatigue, while disengagement describes to what extent an individual experiences distance from their work, work goal and work content. Responses were given on a four-point Likert scale, 1 = “totally agree” to 4 = “totally disagree.” Occupational burnout (OB) was calculated by taking the average of all item responses. The higher score indicates higher burnout. In this study, the Cronbach’s alpha for OLBI was 0.84.

#### Organizational Constraints

The Polish adaptation (63) of the 11-item Organizational Constraints Scale (OCS) was applied, which measures the extent to which participant’s experienced organizational constraints at work (30). Items include situations that interfere with job performance such as inadequate training, poor equipment or supplies, and inadequate help from others. Respondents are asked to indicate how often it is difficult or impossible to do their job because of each item. The range of response options is from 1 (less than once per month, or never) to 5 (several times per day). All items are summed up into a total score. In this study, the Cronbach’s alpha of the scale was 0.88.

#### Interpersonal Conflict at Work

The Polish adaptation (63) of 4-item Interpersonal Conflict at Work Scale (ICAWS) was used (30). The scale measures the participants’ perceived level of interpersonal conflict at work. The items ask how well the employee gets along with others at work, especially in terms of conflicts with others and how often others act negatively to the respondent. Responses were given on a five-point scale, from 1 (less than once per month or never) to 5 (several times per day). The total score is the sum of all items. The Cronbach’s alpha for the ICAWS in this study was 0.81.

#### Perceived Organizational Support

Perceived organizational support (POS) has been estimated by using the Polish adaptation (64) of two subscales, i.e., *Rewards* (four items) and *Fairness* (six items) from Areas of Worklife Survey (AWS) developed by Leiter and Maslach (65). POS is considered as the general perception of being valued by their employer. The reward area addresses the extent to which rewards – monetary, social, and intrinsic – are consistent with the expectations of managers. Fairness is the extent to which decisions at work are perceived as being fair and people are treated with respect (65). Perceived organizational support was calculated by taking the sum of all item responses from both scales. This dimension was successfully applied previously in the study of Sikora (66). Responses were given on a five-point scale, 1 = “totally disagree” to 5 = “totally agree.” The Cronbach’s alpha for POS in the current study was 0.78.

#### Coping With Workload

The Polish adaptation of the 6-item subscale *Workload* from Areas of Worklife Survey (AWS) by Leiter and Maslach (65) was employed. In the Polish version of the tool, the scale has been applied to measure coping with workload, which describes the subjective feeling of an employee with regard to what extent he/she is able to cope with job responsibilities which are perceived by an individual to be consistent (e.g., “I do not take problems from work to my private life”). Responses were given on a five-point scale, 1 = “totally disagree” to 5 = “totally agree.” A higher score indicates a greater ability to cope with a workload. This variable was applied successfully in the previous study of Sikora (66). The Cronbach’s alpha for this dimension in the current study was satisfactory (0.73).

#### Surface Acting

In order to measure surface acting, we utilized two subscales of the Polish adaptation (67) of the revised Emotional Labor Scale (ELS) developed by Lee and Brotheridge (68). The ELS measures deep acting and two aspects of surface acting (hiding feelings and faking emotions). Surface acting refers to the efforts invested in managing the visible aspects of emotions that appear on the “surface,” whereas deep acting refers to the efforts spent in regulating deeply felt emotions. The Polish adaptation of the tool (67) demonstrated that two subscales of surface acting are mutually interrelated, therefore we decided to treat them as one dimension, namely, those which have been confirmed in the Polish version of the tool, which, in turn significantly differ from deep acting. Responses were given on a five-point scale, where 1 = “never” and 5 = “always.” The total score is the sum of six responses derived from both subscales of ELS. A higher score indicates greater surface acting. The Cronbach’s alpha for surface acting in the current study was 0.84.

### Statistical Analyses

The analyses of the study data were conducted using the IBM SPSS version 27.0 for Windows. The general characteristics of participants and job demands, such as organizational constraints (OC), interpersonal conflict at work (ICAW); job resources – perceived organizational support (POS), coping with workload (CWW), as well as surface acting (SA) and occupational burnout (OB) were analyzed using descriptive statistics. Correlations were verified by Pearson’s correlation coefficients. To verify the mediating effects of SA in the association between JD-R and burnout, Hayes’ PROCESS macro 4.0 programme was used. Analysis of mediating effects was performed by inputting Model 4, 95% confidence interval (CI), and 5,000 as the bootstrap sample size. The moderated direct effect of CWW on the relationship between OC and OB through SA was tested by using PROCESS macro (Model 5). Finally, all analyses were controlled for the socio-demographics of nurses, including the work experience, workplace, education level, marital status and number of children. A statistically significant mediating effect was confirmed if the CI of the indirect path did not include 0.

**FIGURE 1:**
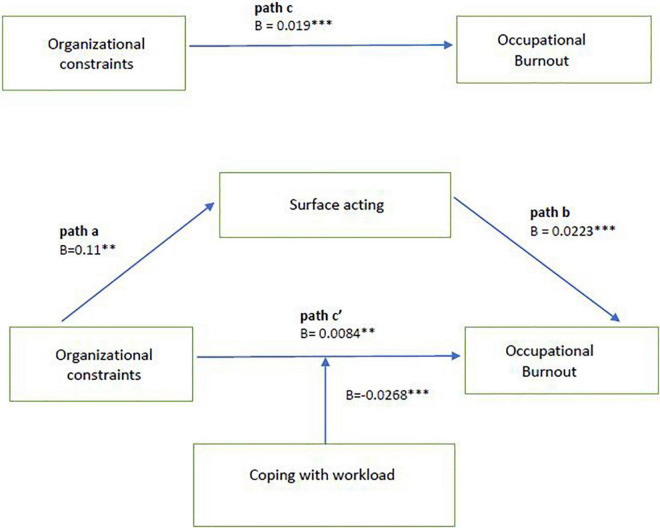
Moderation of direct effect in mediational model. ^**^p < 0.01; ^***^p < 0.001. Model with covariates included (work experience, workplace, education level, marital status, and number of children). B, unstandardized coefficient. Path a, the effect of organizational constraints on surface acting. Path b, the effect of surface acting on occupational burnout; Path c, the total effect of organizational constraints on occupational burnout. Path c’, the direct effect of organizational constraints on occupational burnout after introduction of mediating variable.

The Shapiro-Wilk normality test was applied to consistency of the residual (error) distribution with the normal distribution for each linear model. In the model diagnostics, we also checked the occurrence of multicollinearity between independent variables, based on tolerance (≥0.1 and variance inflation factor (VIF) < 10. Moreover, the Durbin–Watson value was used to assess autocorrelation of errors (result close to 2.00, indicate no problem with the autocorrelation of errors). To avoid multicollinearity problems in model with moderation (Figure 1) we applied mean centering for OCS and CWW. *P*-values less than 0.05 were considered statistically significant.

## Results

### Sample Characteristics

A group of 270 participants took part in the study. As shown in Table 1, all respondents were women and their mean age was 37 years (*SD* = 12.2). Most of the participants were in a relationship (71.5%) – both in terms of marital or informal ties. It is worth mentioning that this group is divided into half in terms of the participants’ number of children – 51.1% did not have any child, while 37% had 1 or 2 children. Another important characteristic of the research group is their educational level – the vast majority of respondents obtained a higher degree diploma (88%). In terms of work experience, less than half of the sample (41.1%) had worked in the nursing profession for at least 16 years, while a quarter of the research group had worked as a nurse for no more than 2 years.

**TABLE 1:** Demographic information of nurses (*N* = 270).

Characteristics *n* (%)	
**Gender**	
Female	270 (100)
**Relationship status**	
Single	77 (28.5)
In relationship	193 (71.5)
**Number of children**	
0	138 (51.1)
1–2	100 (37.0)
3 and more	32 (11.9)
**Education**	
Higher (MA or PhD)	127 (47.0)
Higher (BA)	111 (41.1)
Secondary	32 (11.9)
**Work experience (years)**	
0–2	69 (25.6)
3–15	90 (33.3)
16 and more	111 (41.1)
**Work place**	
OP/ER	36 (13.3)
ICU	53 (19.6)
Other	181 (67.0)

*OP, operating block; ER, emergency room; ICU, Intensive Care Unit.*

In Table 2 was presented burnout level according to general sociodemographic characteristics of participants. The higher level of burnout was reported among nurses without higher education degree, working between 3 and 15 years, not having children and employed in Operating Block/Emergency Room unit.

**TABLE 2:** Burnout according to general characteristics of participants.

		Burnout				
Education	*N* (%)	Mean (SD)	Median (IQR)	Min–Max	*t* or F (df)	*p*
Higher (MA + PHD)	127 (47.0)	2.47 (0.40)	2.44 (0.56)	1.56–3.69	0.749 (2)	0.474
Higher (BA)	111 (41.1)	2.46 (0.38)	2.44 (0.50)	1.50–3.75		
Secondary	32 (11.9)	2.55 (0.41)	2.50 (0.55)	2.00–3.50		
**Work experience (*years*)**						
0–2 years	69 (25.6)	2.45 (0.39)	2.44 (0.63)	1.50–3.75	0.392 (2)	0.676
3–15 years	90 (33.3)	2.50 (0.40)	2.50 (0.63)	1.63–3.69		
16 years and more	111 (41.1)	2.46 (0.39)	2.44 (0.44)	1.50–3.50		
**Children (*number*)**						
0	138 (51.1)	2.51 (0.38)	2.50 (0.50)	1.50–3.75	1.796 (2)	0.168
1–2	100 (37.0)	2.42 (0.39)	2.44 (0.44)	1.56–3.50		
3 and more	32 (11.9)	2.46 (0.41)	2.44 (0.50)	1.63–3.38		
**Marital status**						
In relationship	193 (71.5)	2.47 (0.39)	2.44 (0.13)	1.50–3.50	−0.532 (268)	0.595
Single	77 (28.5)	2.49 (0.39)	2.50 (0.56)	1.50–3.75		
**Workplace**						
OP/ER	36 (13.3)	2.61 (0.45)	2.53 (0.55)	1.88–3.75	2.850 (2)	0.060
ICU	53 (19.6)	2.44 (0.43)	2.50 (0.66)	1.50–3.25		
Other	181 (67.0)	2.46 (0.36)	2.44 (0.50)	1.56–3.50		

*OP, operating block; ER, emergency room; ICU, Intensive Care Unit.*

The next stage of the analysis was to measure the descriptive statistics in the case of all the variables. The results are presented in Table 3. We found that our research group was characterized by moderate levels of burnout (*M* = 2.47; *SD* = 0.38) and surface acting (*M* = 14.34; *SD* = 4.34). Furthermore, the sample reported the average level of job demands, while perceived organizational support (job resource) was relatively high (*M* = 30.64; *SD* = 5.62). Pearson correlations analyses showed that both organizational constraints and interpersonal conflict at work were positively associated with burnout (*r* = 0.40, *p* < 0.01) and surface acting, i.e., OC (*r* = 0.23, *p* < 0.01), ICAW (*r* = 0.30, *p* < 0.01). By contrast, perceived organizational support and coping with workload had a negative correlation with burnout, i.e., POS (*r* = −0.47, *p* < 0.01), CWW (*r* = −0.43, *p* < 0.01), as well as with surface acting, i.e., POS (*r* = −0.21, *p* < 0.01), and CWW (*r* = −0.28, *p* < 0.01).

**TABLE 3:** Means, standard deviations, and Pearson’s coefficient correlations between observed variables (*N* = 270).

Variable	Mean	SD	1	2	3	4	5	6
(1) Organizational constraints (OC)	21.63	7.77	–	0.50**	−0.46**	−0.45**	0.40**	0.23**
(2) Interpersonal conflict at work (ICAW)	7.76	2.70	–	–	−0.48**	−0.33**	0.40**	0.30**
(3) Perceived organizational support (POS)	30.64	5.62	–	–	–	0.32**	−0.47**	−0.21**
(4) Coping with workload (CWW)	16.67	4.13	–	–	–	–	−0.43**	−0.28**
(5) Occupational burnout (OB)	2.47	0.38	–	–	–	–	–	0.39**
(6) Surface acting (SA)	14.34	4.34	–	–	–	–	–	–

**p < 0.01.

### Testing for Mediating Effects

Table 4 presents the fact that organizational constraints had a significant positive effect on burnout (95% CI = [0.014, 0.025], *t* = 6.572, *p* < 0.001). When the mediating variable surface acting was put into the model with covariates, the direct effect of OC on OB was still significant (95% CI = [0.011, 0.022], *t* = 5.694, *p* < 0.001), indicating that SA partially mediated the association between OC and OB. The bootstrapped standardized indirect effect on the path from OC to OB through SA was significantly different from 0 (95% CI = [0.001, 0.006]).

**TABLE 4:** Mediating effect of surface acting (SA) in association between organizational constraints (OC) and burnout (OB).

	Unstandardized coefficient (B)	Standard error (SE)	*t*	*p*	LLCI	ULCI
OC → OB *(total effect)*^a^**	0.019	0.003	6.572	<0.001	0.014	0.025
OC → SA	0.113	0.035	3.259	0.001	0.045	0.181
SA → OB	0.028	0.005	5.644	<0.001	0.018	0.038
OC → OB *(direct effect)*^b^**	0.016	0.003	5.694	<0.001	0.011	0.022
OC → SA → OB	0.003	0.001			0.001	0.006

*Model with covariates included (work experience, workplace, education level, marital status, and number of children). LLCI, the lowest value of the confidence interval; ULCI, the highest value of the confidence interval; OC, organizational constraints; OB, occupational burnout; SA, surface acting.*

*^a^Total effect of organizational constraints on occupational burnout.*

*^b^The direct effect of organizational constraints on occupational burnout after introduction of surface acting.*

Table 5 shows that the positive effect of interpersonal conflict at work on burnout was significant (95% CI = [0.039, 0.072], *t* = 6.538, *p* < 0.001). The direct path between ICAW and OB remained significant (95% CI = [0.027, 0.060], *t* = 5.2140, *p* < 0.001) when SA and the covariates were included in the model, indicating the partial mediation of SA in the association between ICAW and OB. Bootstrapping results showed an indirect effect of 0.0116 (95% CI = [0.004, 0.021]) through surface acting.

**TABLE 5:** Mediating effect of surface acting (SA) in association between interpersonal conflict at work (ICAW) and burnout (OB).

	Unstandardized coefficient (B)	Standard error (SE)	*t*	*p*	LLCI	ULCI
ICAW → OB *(total effect)*^a^**	0.055	0.008	6.538	<0.001	0.039	0.072
ICAW → SA	0.434	0.097	4.477	<0.001	0.243	0.625
SA → OB	0.027	0.005	5.202	<0.001	0.017	0.037
ICAW → OB *(direct effect)*^b^**	0.044	0.008	5.214	<0.001	0.027	0.060
ICAW → SA → OB	0.012	0.004			0.004	0.021

*Model with covariates included (work experience, workplace, education level, marital status, and number of children). LLCI, the lowest value of the confidence interval; ULCI, the highest value of the confidence interval; ICAW, interpersonal conflict at work; OB, occupational burnout; SA, surface acting.*

*^a^The total effect of interpersonal conflict at work on occupational burnout.*

*^b^The direct effect of interpersonal conflict at work on occupational burnout after introduction of surface acting.*

Table 6 displays that perceived organizational support had a significant negative effect on burnout (95% CI = [−0.040, −0.024], *t* = −7.855, *p* < 0.001). When surface acting with covariates were added, the direct effect of POS on OB was still significant (95% CI = [−0.035, −0.020], *t* = −7.075, *p* < 0.001), indicating partial mediation. The indirect effect of POS on OB through SA was significantly different from 0 (95% CI = [−0.008, −0.001]). These findings support hypotheses 1, 2, 3, and 4.

**TABLE 6:** Mediating effect of surface acting (SA) in association between perceived organizational support (POS) and burnout (OB).

	Unstandardized coefficient (B)	Standard error (SE)	*t*	*p*	LLCI	ULCI
POS → OB *(total effect)*^a^**	−0.032	0.004	−7.855	<0.001	−0.040	−0.024
POS → SA	−0.153	0.049	−3.140	0.001	−0.249	−0.057
SA → OB	0.027	0.050	5.634	<0.001	0.018	0.037
POS → OB *(direct effect)*^b^**	−0.028	0.040	−7.075	<0.001	−0.035	−0.020
POS → SA → OB	−0.004	0.002			−0.008	−0.001

*Model with covariates included (work experience, workplace, education level, marital status and number of children). LLCI, the lowest value of the confidence interval; ULCI, the highest value of the confidence interval; POS, perceived organization support; OB, occupational burnout; SA, surface acting.*

*^a^The total effect of perceived organization support on occupational burnout.*

*^b^The direct effect of perceived organizational support on occupational burnout after introduction of surface acting.*

### Testing for Moderation of Direct Effect in Mediational Model

**FIGURE 2:**
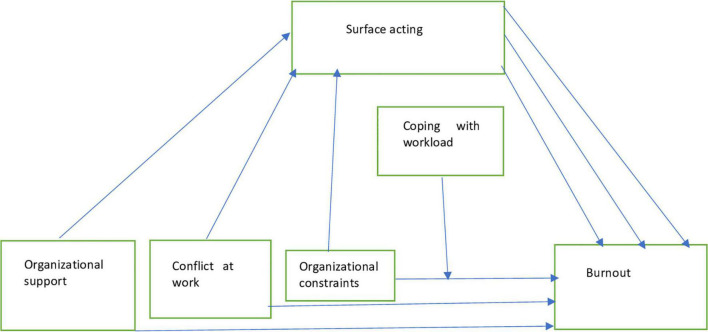
Research model.

In our research model (Figure 2) we hypothesized that coping with workload would moderate a direct path in the mediation process of organizational constraints on burnout through surface acting. Figure 1 shows the results of this analysis using Model 5 of PROCESS macro by Hayes (69). After coping with workload with covariates were put into the model, organizational constraints had a significant positive effect on burnout (95% CI = [0.0023, 0.0145], *t* = 2.7188, *p* < 0.001), and the product of organizational constraints and coping with workload had negative significant effect on burnout (95% CI = [−0.0025, −0.0002], *t* = −2.2331, *p* = 0.02), indicating that CWW moderated the direct effect of OC on OB.

**FIGURE 3:**
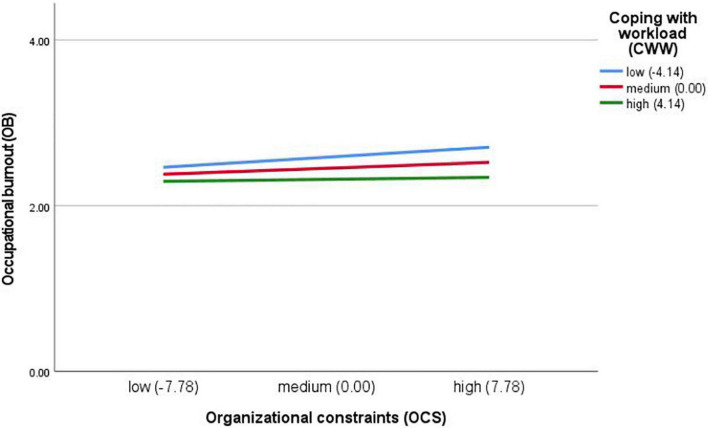
Coping with workload (CWW) as a moderator between organizational constraints (OCS) and occupational burnout (OB). Model with covariates included (work experience, workplace, education level, marital status, and number of children). For participants with high CWW level, OC had no significant effect on burnout. As the CWW decreased to the medium and low level, role of OC had a positive effect on burnout and the effect was increasing gradually, indicating that with the improvement of CWW level, the positive effect of OC on burnout was decreasing gradually.

Further simple slope analysis results are shown in Figure 3. For participants with high coping with workload level (M + 1SD), organizational constraints had no significant effect on burnout (*b*_*simple*_ = 0.003, *t* = 0.670, *p* = 0.504). As the coping with workload decreased to the middle (M) and low level (M-1SD), role of organizational constraints had a positive effect on burnout and the effect was increasing gradually (middle level: *b*_*simple*_ = 0.009, *t* = 2.901, *p* = 0.004; low level: *b*_*simple*_ = 0.016, *t* = 4.416, *p* < 0.001), indicating that with the improvement of coping with workload level, the positive effect of organizational constraints on burnout was decreasing gradually. These findings confirm hypothesis 5.

### Comparison of the Mediating Effects of Surface Acting

The mediating effect of surface acting in the relation between OC and OB was 16% (0.0032/0.0194), while the mediating effect of SA in the relation between ICAW and OB was 21% (0.0116/0.0551). In turn, the mediating effect of SA in the relation between POS and OB was 13% (−0.0042/−0.0317). This implied that interpersonal conflict at work had more of an influence on the burnout of nurses through surface acting compared with the other two analyzed associations.

## Discussion

This study explored how job demands and resources affected burnout among nurses and whether these relationships are mediated by surface acting and moderated by coping with workload. Our findings turned out to be consistent with the JD-R theory, thus confirming hypotheses 1 and 2.

The health-impairment process was indicated by the significant positive association between organizational constraints, interpersonal conflict at work and burnout among nurses. These findings are consistent with previous studies supporting the positive relationship between job demands and burnout in terms of both organizational constraints (26, 70) and conflicts at work (35, 71). As our results pointed out, interpersonal conflicts are stronger significantly associated with burnout. It could be explained by notion that conflicts at work, which are situations that prevent employees from translating ability and effort into high levels of job performance, are associated with strong negative emotions, such as anxiety, hostility or frustration, leading to low job satisfaction and organizational deviance and, consequently burnout (72). In addition, this finding seems to confirm the assumptions of the theory of social relations (36) in which interpersonal conflicts with coworkers and supervisor are related to poor personal psychological outcomes of an employee. Moreover organizational constraints turned out to be also significantly associated with higher level of burnout among nurses. As previous studies stated, a lack of adequate resources including the lack of supplies, lack of equipment, broken equipment and unavailable resources have been shown to be among the top workplace stressors in several countries (73), leading to occupational burnout among the nursing personnel (74).

With respect to the relationship between organizational support considered to be a job resource and burnout, we found its significant negative association. This finding is in line with a previous study according to which the perception of a pleasant working environment and the sense of receiving organizational care could reduce burnout among medical staff (50). Although the relationship between JD-R and burnout is well established in subject-related literature (22), most studies were conducted in the Western countries, whereas our results show that this association is consistent outside the Western context as well.

### The Mediating Role of Surface Acting

Regarding hypotheses 3 and 4, our study demonstrated that surface acting has a partial mediating effect on the positive relationship between both analyzed job demands and organizational support and burnout. In other words, higher organizational constraints and conflicts at work are associated with an increase in surface acting, which is in turn associated with an increase in burnout. This finding is consistent with a previous study of Gilardi et al. (25) in which, using the JD-R model, they confirmed the mediating role of surface acting between job demands and burnout among Italian nurses. It could be explained by notion that the nurses’ efforts to manage their emotions that are specifically triggered by the lack of equipment, as well as interpersonal conflicts at the workplace activate a resource depleting process, which leads to emotional exhaustion and the adoption of a strategy of disengagement. We assume that both these stressful situations provoke emotional dissonance because the negative emotions which emerge are appraised as inappropriate for the interiorized role of a health care worker. This dissonance generates a state of tension because nurses experience a person–role conflict (48). If this state of tension persists, it leads to the depletion of the resource of self-control, and, consequently, mental and physical exhaustion (75).

By contrast, as demonstrated, a higher level of perceived organizational support is related to a decrease in surface acting, which in turn is associated with a decrease in burnout among Polish nurses. Several explanations for such an effect could be proposed. As Chou et al. (49) showed, when supervisor support is available, nurses are more capable of availing of deep acting which, in turn, is associated with a decrease in occupational burnout among a professional group of nurses (76). Goussinsky and Livne (77) suggested that support from the supervisor - a key component of organizational support - encourages the internalization of work roles and leads workers to engage more in deep acting. On the other hand, nurses who reported low levels of support are more likely to regulate their emotions using surface acting which was found to be a significant predictor of burnout (49).

### The Moderating Role of Coping With Workload

With respect to hypothesis 5, our study indicates that coping with workload moderated the direct path in the mediation process of organizational constraints on burnout through surface acting among Polish nurses. This is consistent with the buffering impact of job and personal resources on the relationship between job demands and burnout postulated by the JD-R model (21). In the case of high organizational constraints, nurses characterized by greater abilities to cope with workload reported lower burnout compared with nurses with a decreased level of CWW. This finding is in accordance with the study of Chang and Chan (78) conducted among Taiwanese nurses that examined the buffering effect of proactive coping with relation to burnout. In line with this study (78), we assume that individuals with a greater capacity to cope with workload, considered by us to be a kind of proactive coping, are future-oriented and know how to utilize the acquired resources to face distress before they begin to feel emotionally exhausted. In the case of workload which is a big stressor, proactive nurses often make efforts to plan in advance which enables them to deal more easily with multiple work responsibilities. Similarly, it could be assumed that in the case of organizational constraints, they are more likely to build up resources that serve as buffers against stress deriving from, e.g., inadequate training or lack of supplies, and consequently, against the occurrence of burnout.

It is worth mentioning a few contributions of our study. First, to the best of our knowledge, so far no studies have investigated the mediating role of surface acting between organizational constraints and interpersonal conflict at work (job demands) which are regarded as very important stressors among nursing staff (28, 29). Thus, our study filled this gap in subject-related literature by using the JD-R framework. Second, we pointed out, that not only stressors related to interpersonal relations are associated with surface acting, and consequently, with higher burnout among nurses (25), but also organizational constraints have similar detrimental effect. Third, we included coping with workload considered as a personal resource which turned out to be significant moderator in relationship organizational constraints – burnout. To the best of our knowledge, this is the first study exploring the issue of coping with workload as a moderating variable in the association between job demands and burnout. This finding has practical implications by emphasizing the importance of psychological training which should enhance the capability of coping with workload in this professional group. Fourth, our study supported validity of the Job Demands-Resources theory also in Eastern Europe, while most studies using this theoretical framework have been conducted in Western countries. Fifth, since there is insufficient amount of research carried out during the late wave of COVID-19 pandemic, our study could be regarded as a contribution to the current knowledge regarding mental health of nurses.

### Limitations and Future Directions

Although the results of the study deepen our understanding concerning the mediating and moderating effects in associations between job demands and burnout among nurses from Central-Eastern Europe, there are several limitations which should be listed. Firstly, our sample consisted of nurses from only one hospital and only females, therefore future studies may take into account a more representative sample of nurses to draw better conclusions about JD-R, burnout and surface acting. We recommend conducting studies on bigger samples and analyse nurses, including both females and males, recruited from many Polish hospitals, located both in big cities, as well as small cities. Secondly, since we analyzed cross-sectional data, our results should be interpreted with caution, as we reported associations between job demands, resources and burnout, yet we cannot confirm causal relations. This limitation may be addressed by the collection and analysis of longitudinal data, e.g., data obtained during pandemic and in post-pandemic period. Thirdly, in our research model we did not include any personal traits, such as, for instance self-efficacy or ego-resiliency, which, according to research, affect JD-R and burnout among nurses (23, 25). Thus, in the future it may be worth exploring personal traits and their role with relation to burnout in order to get a more comprehensive overview of the well-being of Polish nurses. Since these variables were not included in our model, they may have unknown effects in terms of the estimates from our analysis. Fourthly, all study variables were measured using self-report questionnaires, which may lead to potential bias and could affect the accuracy of the assessment, thus future studies may combine self-report questionnaires with more objective measurements.

## Conclusion

This is the first study relating to the burnout level of Polish nurses during the COVID-19 pandemic. The findings enrich our knowledge on the mediating and moderating mechanisms in order to explain the association between job demands, resources and burnout among nurses during the latest wave of the COVID-19 pandemic. We confirmed that job demands, such as interpersonal conflict at work and organizational constraints significantly increase burnout, both directly and indirectly by means of surface acting, whereas, job resources, such as organizational support, decrease it. Moreover, perceived organizational support has a direct negative impact on burnout, as well as an indirect effect via a decrease in surface acting.

Our results suggest that when nurses experience high job demands, strengthening organizational support and coping with workload could help alleviate burnout. It is important especially in the context of COVID-19 pandemic, during which many nurses perceived their workplace as potentially harmful and dangerous. As previous studies reported, organizational support during COVID-19 pandemic was associated with lower level of anxiety (79), lower burnout occurrence (50) and increased work engagement among nurses (80). Moreover, as we showed, organizational support could reduce burnout in nurses via a decrease in surface acting. Thus, interventions that provide effective emotional regulation management education, as well as psychological training concerning adequate coping strategies could help reduce or prevent the occurrence of burnout among nurses as well as decrease the negative effect of insufficient perceived organizational support. In the context of the COVID-19 burden and its mental consequences, it is worth mentioning that hospital organizations that nurses work in should provide organizational support, for instance, work allocation and flexible work hours, in order to alleviate burnout among this professional group (81).

## Data Availability Statement

The original contributions presented in this study are included in the article/supplementary material, further inquiries can be directed to the corresponding author.

## Ethics Statement

This study was reviewed and approved by the Jagiellonian University Bioethics Committee (protocol code: 1072.6120.298.2021, approval date: 15.12.2021).

## Author Contributions

GW: research idea, research design, conceptualization, literature review, data collection, data interpretation, draught manuscript, and revision of work. AW: conceptualization, project administration, work supervision, revision of work, and funding. IB: data interpretation, visualization, and revision of work. All authors contributed to the article and approved the submitted version.

## Conflict of Interest

The authors declare that the research was conducted in the absence of any commercial or financial relationships that could be construed as a potential conflict of interest.

## Publisher’s Note

All claims expressed in this article are solely those of the authors and do not necessarily represent those of their affiliated organizations, or those of the publisher, the editors and the reviewers. Any product that may be evaluated in this article, or claim that may be made by its manufacturer, is not guaranteed or endorsed by the publisher.
